# Comparación del ángulo de la guía condílea sagital obtenida del registro radiográfico y clínico en pacientes dentados

**DOI:** 10.21142/2523-2754-0902-2021-060

**Published:** 2021-06-21

**Authors:** Richard Jesús Ñahuincopa-Ríos, Paul Marcelo Ñahuincopa-López, Rubén Gerardo Ángeles-López

**Affiliations:** 1 División de Rehabilitación Oral, Universidad Científica del Sur. Lima, Perú. richardnahuincoparios@gmail.com, rubangeles76@yahoo.es Universidad Científica del Sur División de Rehabilitación Oral Universidad Científica del Sur Lima Peru richardnahuincoparios@gmail.com rubangeles76@yahoo.es; 2 Carrera de Estomatología, Universidad Nacional San Luis Gonzaga de Ica (Unica). Ica, Perú. Universidad Nacional San Luis Gonzaga Carrera de Estomatología Universidad Nacional San Luis Gonzaga de Ica (Unica) Ica Peru; 3 Carrera de Estomatología, Universidad Científica del Sur. Lima, Perú. marelo53@hotmail.com Universidad Científica del Sur Carrera de Estomatología Universidad Científica del Sur Lima Peru marelo53@hotmail.com

**Keywords:** cefalometría, radiografía panorámica, cóndilo mandibular, oclusión dental, Cephalometry, Panoramic radiography, Mandibular condyle, Dental occlusion

## Abstract

**Objetivo:**

Comparar el ángulo de la guía condílea sagital obtenida del registro radiográfico y clínico en pacientes dentados.

**Materiales y métodos:**

La muestra estuvo conformada por un grupo único de estudio de 32 pacientes, en el que se evaluó la radiografía lateral estricta y los registros posicionales: en relación céntrica, registro protrusivo a 5 mm en lateralidad derecha y registro protrusivo a 5 mm en lateralidad izquierda. Con el registro posicional del arco facial se articuló el modelo superior, con el registro posicional en relación céntrica se articuló el modelo inferior, con los registros laterales protrusivos de los lados derecho e izquierdo se obtuvo la medida del ángulo de la guía condílea sagital para la programación del articulador semiajustable. El plan estadístico en la presente investigación utilizó el programa SPSS versión 24, la normalidad fue evaluada usando el test de Shapiro-Wilk, también se realizaron las pruebas de T de Student y correlación de Pearson.

**Resultados:**

Se determinó estadísticamente que el género y la edad influyen en la medida del ángulo de la guía condílea sagital. El método radiográfico presentó un ángulo de guía condílea de 35,69 ±5,18 y con el método clínico fue 35,69 ± 5,16 (p > 0,05). La prueba de correlación de Pearson sí mostró una correlación importante entre ambos métodos r = 0,948, p < 0,001.

**Conclusiones:**

Existe alta correlación en las medidas obtenidas del ángulo de la guía condílea sagital con los registros radiográficos y clínicos; esta concordancia permitiría reemplazar un método por el otro.

## Abstract (en)

**Objective:**

To compare the angle of the sagittal condylar guidance obtained from the radiographic and clinical records of dentate patients.

**Materials and Methods:**

The sample consisted of 32 patients in whom strict lateral radiography and positional records were performed: in centric relation, protrusive recording 5 mm in right laterality and protrusive recording 5 mm in left laterality. With the positional registration of the facebow, the upper model was articulated, while the lower model was articulated with the positional registration in centric relation, and with the protrusive lateral registrations on the right and left side the condylar guidance was obtained for programming the semi-adjustable articulator. Statistics were performed with the SPSS program version 24 in Spanish. Normality was evaluated using the Shapiro-Wilk test, for data with normal distribution, and the Student's T test and Pearson's correlation were performed in descriptive statistics.

**Results:**

The statistical analyses showed that gender and age influence the angle measurement of the sagittal condylar guide. The radiographic method presented a condylar guide angle of 35.69 ± 5.18, being 35.69 ± 5.16 with the clinical method (p> 0.05). Pearson's correlation test showed an important correlation between the two methods (r = 0.948, p <0.001).

**Conclusions:**

There is a high correlation in the measurements obtained from the angle of the sagittal condylar guide and the radiographic and clinical records, indicating that both methods are effective.

## INTRODUCCIÓN

La guía condílea sagital (GCS) es uno de los factores intervinientes en el establecimiento de la oclusión con influencia directa en la morfología oclusal. Cuando el componente cóndilo mandibular-disco articular sale de la posición de relación céntrica e inicia su recorrido por la eminencia articular en un movimiento de protrusión mandibular, se va formando un ángulo conforme se aparta del plano horizontal de Frankfort, denominado ángulo de la guía condílea sagital (AGCS). La GCS es considerada un factor fijo y se mantiene estable en el paciente sano ^1^.

Balkwill ^2^, en 1864, describió la dificultad de determinar la inclinación de la GCS en el ser humano vivo, diseñó un dispositivo y estimó el AGCS en 26°; Isaacson ^3^, en su estudio clínico de la trayectoria condílea del año 1959, consideró a la GCS de vital importancia en el inicio de la rehabilitación oral del paciente. Christensen *et al*. ^4^, en 1979, con base en estudios anatómicos, radiográficos y cinerradiográficos correlacionó la GCS y el AGCS en relación con la programación de los articuladores ajustables. Donegan *et al*. ^5^, en 1991, halló en sus estudios una simetría bilateral en el valor del AGCS en 31° y utilizó para este fin los registros interoclusales protrusivos (RIP). Prasad *et al*. ^6^, en 2012, correlacionó la GCS obtenida mediante los RIP en cera y los métodos de trazado en radiografías panorámicas (RP) de pacientes dentados e ideó un método de trazado sobre la radiografía panorámica, con el cual delineó la inclinación de la GCS relacionándolo con el trazado del plano de Frankfort (PF) para determinar el AGCS radiográfico, los resultados determinaron una correlación positiva fuerte entre el método clínico de los RIP y el método radiográfico, presentando mínimas diferencias los valores obtenidos, concluyendo que el AGCS radiográfico obtenido puede ser utilizado en la programación de la GCS del articulador semiajustable (ASA). Galagali *et al*. ^7^, en 2016, evaluó y comparó la correlación entre el AGCS obtenido del RIP con los modelos montados en el ASA, los trazados realizados en la radiografía lateral estricta (RLE) y en la RP de pacientes dentados de 20 a 40 años, lo que demostró estadísticamente una mayor correlación positiva del RIP con los trazos realizados en la RLE y concluyeron que la RLE es confiable para el registro del AGCS. Paul *et al*. ^8^, en 2017, realizó su estudio en pacientes desdentados de 45 a 75 años, utilizando el RIP con los modelos montados en el ASA frente al trazado en las RLE y RP, con lo que hallaron una correlación positiva estadísticamente significativa entre los tres métodos y concluyó que los valores obtenidos del trazado cefalométrico se pueden usar como complemento al método clínico, pero no de forma independiente en la programación del ASA. Dewan *et al*. ^9^, en 2019, realizó un estudio en una población de Arabia Saudita en pacientes dentados de 20 a 40 años, los resultados indicaron que los valores obtenidos utilizando los RIP con los modelos montados en el ASA fueron menores en comparación con los valores obtenidos con el método radiográfico en las RP, con una diferencia de valores en ambos métodos muy significativas. Mawani *et al*. ^10^, en 2019, tuvieron como objetivo comparar la RP y la tomografía computarizada de haz cónico (TCHC) para establecer la GCS como alternativa en la programación del ASA y articuladores totalmente ajustables (ATA), estudiaron una muestra de 20 varones y 20 mujeres, de 20 a 40 años, y determinaron que no hubo diferencias significativas entre ambas técnicas, pero con el aumento de la edad, los valores obtenidos de la GCS disminuían; por lo tanto, están correlacionados y son comparables para realizar la programación del ASA y ATA. Naqash *et al*. ^11^, en 2020, utilizaron los siguientes métodos: clínico, radiográfico y pantográfico para determinar el AGCS en una muestra de 23 pacientes dentados de 18 a 30 años, y concluyeron que existe una fuerte correlación entre los tres métodos para hallar el AGCS. Das *et al*. ^12^, en 2020, compararon el método clínico con el RIP y el método radiográfico con la TCHC para determinar el AGCS en la programación del ASA y ATA, en una muestra de 20 varones y 20 mujeres, con un rango de edad de 20 a 40 años. Midieron la TCHC a través de un programa computarizado y determinaron que los resultados fueron comparables, con respecto al sexo no hubo diferencia estadísticamente significativa, los valores obtenidos fueron disminuyendo conforme aumentaba la edad, y concluyeron que se puede aprovechar en aquellos pacientes que requieren la TCHC, y realizar la programación del ASA y ATA.

Si asumimos que el tiempo es un bien que no podemos recuperar ni acumular, el propósito de este estudio fue demostrar que el método radiográfico para hallar el AGCS es preciso y confiable, con valores similares a los obtenidos con el método clínico, lo que supone un ahorro de tiempo y recursos considerables en la práctica clínica estomatológica para la programación de la caja condílea en el ASA, punto importante para el inicio de toda rehabilitación oral. Por lo tanto, el objetivo principal de la presente investigación fue determinar la concordancia de las medidas de AGCS, a través de la comparación del registro radiográfico y clínico del ángulo de la guía condílea sagital obtenida en los pacientes dentados que acudieron a la Clínica Dental de Rehabilitación Corazón de Jesús (Huamanga, Perú) durante el periodo comprendido entre 2015 y 2017.

## MATERIALES Y MÉTODOS

El estudio fue de tipo observacional, descriptivo, transversal y retrospectivo ^13^. La población estuvo conformada por 32 pacientes adultos que acudieron a la Clínica Dental de Rehabilitación Corazón de Jesús, en la ciudad de Huamanga en la región Ayacucho, a tomarse la RLE indicada por el especialista en Ortodoncia y Ortopedia Maxilar, obtenidas entre enero del 2015 y diciembre del 2017, y seleccionadas según los siguientes criterios de inclusión: RLE de pacientes que se encuentren entre los 18 y 40 años, de ambos sexos, con dentición permanente, sin tomar en cuenta los terceros molares, y que pueden presentar ausencia de premolares (extraídos por indicación ortodóntica), y pacientes que presentaron clase I molar y canina, sobremordida horizontal entre 2 y 4 mm, y deslizamiento en céntrica con un máximo de 1,5 mm. Se excluyeron las RLE de pacientes que presentaron algún tipo de aparato protésico e implantes, trastornos en la articulación temporomandibular, enfermedad periodontal, restauraciones en molares permanentes, bruxómanos y pacientes con enfermedades sistémicas en general. 

### Muestra y aspectos éticos

El cálculo del tamaño muestral utilizó la fórmula estadística para la comparación de dos proporciones y la muestra estuvo conformada por 32 pacientes que cumplieron con los criterios expuestos. El trabajo fue aprobado por el Comité Institucional de Ética en Investigación de la Universidad Científica del Sur (CIEI-CIENTÍFICA), con el N.° 000401. Las RLE fueron indicadas por el clínico tratante y pertenecían al archivo activo de Historias Clínicas de la Clínica Dental de Rehabilitación Corazón de Jesús. Se procedió a explicar a los pacientes la naturaleza de la investigación, los procedimientos radiográficos y clínicos, y, una vez comprendidos los términos, se obtuvo la participación y aprobación de los pacientes seleccionados, para lo que se procedió a la firma del consentimiento informado y se garantizó el anonimato de los pacientes que integraron la muestra, de acuerdo con los lineamientos de la Declaración de Helsinki ^14^.

### Obtención de las radiografías

Los especímenes seleccionados (RLE) fueron obtenidos con el equipo radiográfico digital de procedencia coreana, de la marca Point Nix, modelo Point 800S HD Plus, a un miliamperaje de 10 mA y kilovoltaje de 70 kV, con una toma aproximada de 17 segundos. El equipo tiene un sensor super Oled que brinda imágenes de mejor resolución con menos del 1% de distorsión, almacenadas en formato digital, procesadas con el programa informático CDX View DICOM ^15^, impresos en la Carestream Dryview 5700 (impresora láser) ^16^.

### Registro radiográfico

**Figura 1:**
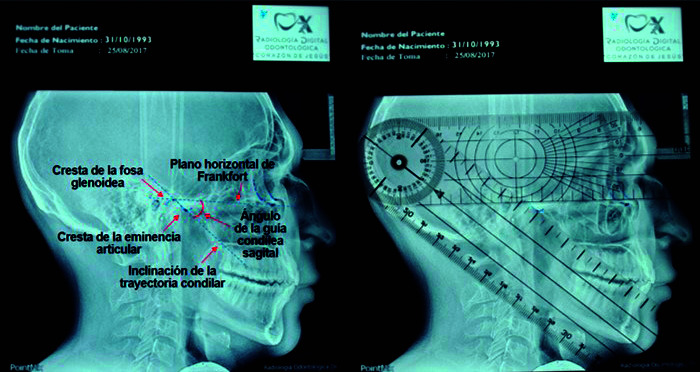
Procedimiento para obtener la medida del AGCS a través del método radiográfico

Seleccionada la muestra, fue clasificada de acuerdo con el orden numérico ascendente del 1 al 32, para luego identificar los puntos craneométricos y los planos referenciales que determinan el AGCS. Se siguió el modelo propuesto en los estudios de Galagali *et al*. ^7^, Prasad *et al*. ^6^, Paul *et al*. ^8^, Singh *et al*. ^17^, Kwon *et al*. ^18^, Kaur *et al*. ^19^ y Shetty *et al*. ^20^. Se utilizó el negatoscopio LED, marca Med Hame, de procedencia china, de 3200 lúmenes, y se posicionó el papel de acetato transparente sobre la RLE. Se identificó y marcó los puntos craneométricos porión (punto más alto en el margen del meato auditivo) y orbital (punto más bajo en el margen de la órbita), los cuales se unieron para el trazado del plano de referencia horizontal de Frankfort; se trazó la cavidad glenoidea y el contorno de la pared posterior de la eminencia articular, se identificó el punto más alto y más bajo, se determinó el plano de inclinación de la trayectoria condilar que se intersecta con el plano de Frankfort, y se conformó el AGCS, que fue medido con el goniómetro (Figura 1).

### Registro clínico

Para el registro del método clínico, se colocó entre los dientes anteriores las láminas del Long ^21^, con el objetivo de separar los dientes posteriores por lo menos 3 mm, por un tiempo de 15 minutos, para inducir la desprogramación de las engramas neuromusculares. Se adiestró al paciente en el reconocimiento del primer punto de contacto y, con la ayuda de un espejo facial, se le hizo ver la magnitud del desplazamiento protrusivo hasta 5 mm y se colocó marcas con lápiz a nivel de la línea media dentaria inferior, con la finalidad de ser un movimiento repetible. Se instruyó al paciente para que desplace la mandíbula 5 mm hacia el lado derecho y la misma distancia hacia el lado izquierdo, y se hizo marcas con lápiz para que pueda repetir el movimiento, pero esta vez ocluyendo y registrando cada una de las tres posiciones en la lámina de cera tipo cavex de arcada completa, compuesta por 2 láminas de cera en el lado de trabajo y 3 láminas de cera en el lado de balance. Se realizó lo mismo en el lado izquierdo y se procedió a marcar los 3 registros interoclusales según el número asignado a cada paciente. 

Se utilizó el ASA tipo arcón de la marca Bio Art, modelo 4000, con el arco facial modelo estándar. Se registró la posición espacial del maxilar superior para su respectivo montaje, se hizo el registro interoclusal con lámina de cera tipo cavex en la posición de relación céntrica, se montó el modelo inferior con el ángulo de la guía condílea en 0° y el ángulo de Bennett en 0°, para su posterior programación, con los modelos montados en el ASA ^22^.

**Figura 2:**
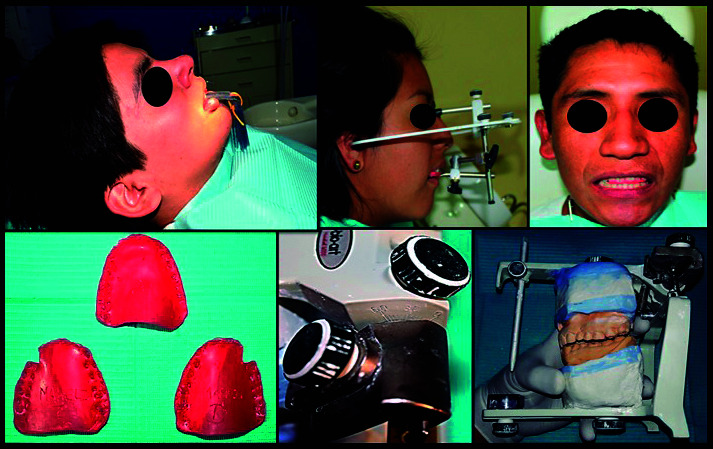
Procedimiento para obtener la medida del AGCS a través del método clínico

Para el registro del AGCS, se aflojaron los tornillos centrales posteriores, se ajustó la guía condílea en 0° y el ángulo de Bennett en 0°, se giró por completo la rama superior del ASA con su respectivo modelo y se colocó cuidadosamente el registro de lateralidad derecha sobre el modelo superior. Se tomó con una sola mano el ASA asegurándose de que haya un asentamiento completo del registro de lateralidad derecha, colocándose el cóndilo derecho del articulador en la caja de la guía condílea derecha, y se observó que el cóndilo izquierdo se había separado de las superficies posterior y superior de su respectiva caja condilar. Se aflojó el tornillo de fijación de la guía condílea izquierda presionando ligeramente hacia abajo la guía condílea hasta que la pared superior tocó el cóndilo izquierdo (sin presión), y se ajustó el tornillo de fijación de la guía condílea en esa posición. Se verificó visualmente que los dientes no se habían separado de sus huellas en el registro lateral de cera, para obtener la desviación lateral (ángulo de Bennett), y se aflojó el tornillo de fijación lateral y movió la guía lateral hasta tocar la pared mesial del cóndilo, ajustando en esa posición el tornillo de fijación; así se obtuvo la medida del AGCS del lado izquierdo y el ángulo de Bennett del mismo lado. Para obtener el AGCS derecho, se ajustó utilizando el registro de cera de lateralidad izquierda y se repitió el procedimiento descrito anteriormente ^22^ (Figura 2).

### Análisis estadístico

Los datos obtenidos con los métodos radiográfico y clínico para el registro del AGCS, sexo y edad fueron analizados mediante el programa IBM® SPSS® Statistics (Chicago, Ill., EE. UU.), versión 24.0, y se realizó el análisis univariado de la variable respuesta (medida del AGCS), con lo que se obtuvo la media y la desviación estándar. Se realizó el análisis bivariado usando la prueba T de Student para muestras independientes y se ejecutó la prueba del coeficiente de correlación de Pearson, lo que estableción un valor “p” para determinar la diferencia estadísticamente significativa.

## RESULTADOS

En la Tabla 1 se observa la distribución de la muestra por sexo y edad. No se encontró diferencia significativa entre hombres y mujeres (p > 0,05). La Tabla 2 permite comparar el valor del AGCS entre ambos métodos. El método radiográfico presentó un ángulo de guía condílea de 35,69 ± 5,18 y con el método clínico presentó 35,69 ± 5,16 (p = 0,100). Finalmente, la tabla 3 muestra la prueba de correlación de Pearson entre ambos métodos, con un valor de correlación directa importante entre ambos métodos r = 0,948, p < 0,001.

**Tabla 1:** Comparación de la edad en la muestra evaluada según sexo.

	Sexo	N	Media	D. E.
Edad	Varón	15	25,47	4,84
	Mujer	17	22,88	4,61

Prueba T de Student p > 0,05

**Tabla 2:** Comparación del ángulo de la guía condílea sagital obtenida del registro radiográfico y clínico de los pacientes dentados evaluados

Variable	N	Media	D. E.
RRX	32	35,69	5,18
RCL	32	35,69	5,16

RRX= Registro radiográfico

RCL= Registro clínico

Prueba T de Student para muestras relacionadas, p = 0,100

**Tabla 3:** Correlación entre las medidas obtenidas del ángulo de la guía condílea sagital de los registros radiográfico y clínico

Variables	RRX
RCL	0,948*
p	<0,001
n	32

RRX = Registro radiográfico

RCL = Registro clínico

*Correlación de Pearson

## DISCUSIÓN

La importancia de identificar con precisión el AGCS para la programación de la GCS mecánica del ASA redundará en la reproducción fidedigna de las dinámicas mandibulares necesarias para la identificación de los puntos prematuros de contacto que pudieran interferir en una oclusión dental balanceada en el proceso de rehabilitación oral. Donegan *et al*. ^5^ utilizaron los RIP y determinaron en sus estudios una simetría bilateral del valor del AGCS en 31°; sin embargo, la practicidad de estos tiempos permitió que diversos estudios de investigación, hayan asignado valores promedio para el AGCS que van desde 22° hasta 65° ^(2, 3, 17, 18, 23-29)^. Los autores de estos artículos difieren de lo afirmado por Donegan *et al*. ^5^ y concuerdan con los demás investigadores al afirmar que es necesaria la personalización del AGCS, tal como precisan Singh *et al*. ^17^ en sus estudios, al afirmar que en pacientes con diversas relaciones esqueléticas asociadas con la oclusión clase I, II y la maloclusión III se obtuvo una mayor heterogeneidad en las medidas del AGCS.

En el caso de los métodos de registro del AGCS, se eligió el RIP, basado en los estudios de Utz *et a*l. ^23^, quienes proporcionaron un grosor adecuado a las láminas de cera para no afectar la exactitud de los registros posicionales. Respecto de la elección del método radiográfico, se optó por los trazados de puntos y planos craneométricos sobre la RLE, lo cual concuerda con las investigaciones de Paul *et al*. ^8^ y Galagali *et al.*^7^, quienes demostraron estadísticamente una mayor correlación positiva del RIP con los trazados realizados sobre la RLE para obtener registros confiables de las medidas del AGCS y como complemento al método clínico en la programación del ASA. Estos autores, a su vez, difieren de los estudios de Prasad *et al*. ^6^, Shreshta *et al*. ^24^, Shah *et al*. ^25^, Khalikar *et al*. ^26^, Kaur *et al*. ^27^ y Tannamala *et al*. ^28^, que utilizaron la RP que induce a la distorsión en la proyección de las imágenes al incorporar una magnificación de 1,2 x. ^(6, 24-28)^, lo que se traduce en valores mayores de 2° a 10° ^(6, 24-28)^ con respecto a los obtenidos con el RIP. Así mismo, Kwon *et al.*^18^ reportaron que los estudios realizados en TCHC fueron un poco más cercanos, pero aún mayores en 5° a 6° a los obtenidos por el RIP.

Con respecto a los resultados obtenidos en la presente investigación, al comparar los promedios de las medidas obtenidas del AGCS a través de los métodos radiográficos y clínicos, se observó que no presentan diferencias en las medidas obtenidas (tabla 2), lo cual coincide con los resultados obtenidos por Shreshta *et al*. ^24^, Galagali *et al*. ^7^ y Paul *et al*. ^8^, pero a la vez difiere de los estudios de Shah *et al*. ^25^, Dewan *et al*. ^9^, Zamacona *et al*. ^29^ y El-Gheriani y Winstanley ^30^, quienes concluyeron que hay variaciones significativas en los valores de las medidas obtenidas del AGCS en ambos lados. Estos resultados se obtienen, tal vez, debido a la pluralidad de las muestras estudiadas a diferencia de la presente investigación que presentó una muestra homogénea de pacientes dentados con respecto a la distribución del sexo y la edad.

Los autores coinciden con Mawani *et al*. ^10^ y Das *et al*. ^12^ al afirmar que los valores obtenidos por el método radiográfico del AGCS tienden a disminuir con el avance de la edad; otro aspecto a tomar en cuenta es la escala numérica en la guía condílea del ASA, la cual se encuentra graficada con aumentos de 5°. Para una programación más precisa, esta escala debería incrementarse de 1° en 1°, un factor importante que deberían tener en cuenta los fabricantes de los ASA. 

Adicionalmente, se recomienda incorporar, en posteriores investigaciones, programas computarizados como los visualizadores en dos y tres dimensiones para la ubicación de los puntos craneométricos, el trazado de los planos de referencia y la medición del AGCS, a fin de eliminar las inexactitudes que puedan presentarse durante el proceso manual. Se recomienda también ampliar el tamaño de la muestra e incorporar variables adicionales como pacientes edéntulos parciales y totales, o incluir información acerca del lado de preferencia al masticar, para determinar su influencia en el complejo cavidad glenoidea-cóndilo articular, así como utilizar diferentes ASA, ATA y arcos faciales profesionales que tomen como referencia el plano de Frankfort. Finalmente, es conveniente contrastar las medidas obtenidas del AGCS en la toma radiográfica lateral estricta con otros métodos de medición, como el trazador pantográfico Cadiax® Compact, que sirve para el registro computarizado del AGCS utilizado por Naqash *et al*. en su estudio ^11^.

## CONCLUSIONES

Existe una alta correlación entre las medidas obtenidas del ángulo de la guía condílea sagital y los registros radiográficos y clínicos, lo cual permitiría reemplazar un método por el otro.

## Fuente de financiamiento:

Autofinanciado
